# Green tea catechins EGCG and ECG enhance the fitness and lifespan of *Caenorhabditis elegans* by complex I inhibition

**DOI:** 10.18632/aging.203597

**Published:** 2021-10-04

**Authors:** Jing Tian, Caroline Geiss, Kim Zarse, Corina T. Madreiter-Sokolowski, Michael Ristow

**Affiliations:** 1 Department of Human Nutrition, Institute of Nutrition, Friedrich Schiller University Jena, Jena 07743, Germany; 2 MOE Key Laboratory of Environment Correlative Dietology, College of Food Science and Technology, Huazhong Agricultural University, Wuhan 430070, China; 3 Laboratory of Energy Metabolism, Institute of Translational Medicine, Department of Health Sciences and Technology, ETH Zurich, Schwerzenbach 8603, Switzerland; 4 Molecular Biology and Biochemistry, Gottfried Schatz Research Center, Medical University of Graz, Graz 8010, Austria

**Keywords:** aging, reactive oxygen species, mitochondria, polyphenols, *C. elegans*

## Abstract

Green tea catechins are associated with a delay in aging. We have designed the current study to investigate the impact and to unveil the target of the most abundant green tea catechins, epigallocatechin gallate (EGCG) and epicatechin gallate (ECG).

Experiments were performed in *Caenorhabditis elegans* to analyze cellular metabolism, ROS homeostasis, stress resistance, physical exercise capacity, health- and lifespan, and the underlying signaling pathways. Besides, we examined the impact of EGCG and ECG in isolated murine mitochondria.

A concentration of 2.5 μM EGCG and ECG enhanced health- and lifespan as well as stress resistance in *C. elegans*. Catechins hampered mitochondrial respiration in *C. elegans* after 6–12 h and the activity of complex I in isolated rodent mitochondria. The impaired mitochondrial respiration was accompanied by a transient drop in ATP production and a temporary increase in ROS levels in *C. elegans*. After 24 h, mitochondrial respiration and ATP levels got restored, and ROS levels even dropped below control conditions. The lifespan increases induced by EGCG and ECG were dependent on AAK-2/AMPK and SIR-2.1/SIRT1, as well as on PMK-1/p38 MAPK, SKN-1/NRF2, and DAF-16/FOXO. Long-term effects included significantly diminished fat content and enhanced SOD and CAT activities, required for the positive impact of catechins on lifespan.

In summary, complex I inhibition by EGCG and ECG induced a transient drop in cellular ATP levels and a temporary ROS burst, resulting in SKN-1 and DAF-16 activation. Through adaptative responses, catechins reduced fat content, enhanced ROS defense, and improved healthspan in the long term.

## INTRODUCTION

Clinical trials and epidemiological studies have revealed health benefits associated with green tea consumption, including a significant reduction in systolic blood pressure [1] and fasting glucose [2] as well as weight loss in type 2 diabetes patients [3] and in women with central obesity [4]. The most abundant polyphenols in green tea leaves are epigallocatechin gallate (EGCG), epicatechin gallate (ECG), epigallocatechin (EGC), and epicatechin (EC), forming 30–42% of the solid green tea extract [5]. EGCG accounts for roughly 50% and ECG for 20% of the total catechin amount in green tea leaves [6]. A randomized, placebo-controlled clinical trial testing a daily supplementation with 400 mg EGCG confirmed the safety of a one-year administration with EGCG. It revealed further that plasma concentrations of EGCG reached a measurable level after six months [7]. A recent study tested the bioavailability of EGCG combined with various food supplements. After overnight fasting, consumption of 150 mg green tea extracts already resulted in plasma level peaks of 10 ng/ml/kg after 60–180 min [8]. *In vivo* experiments in various model organisms suggested a beneficial effect of green tea catechins on lifespan due to metabolic adaptation and enhanced resistance to reactive oxygen species (ROS). For instance, dietary supplementation with EGCG-rich green tea extracts (10 mg/ml EGCG) affected glucose metabolism and increased health- and lifespan in *Drosophila melanogaster* [9]. Besides, green tea polyphenol-containing water (80 mg/l) extended the lifespan of male C57BL/6 mice [10]. Moreover, treatment of *Caenorhabditis elegans (C. elegans)* with EGCG at concentrations of 50–300 μM during early-to-mid adulthood promoted lifespan, and 200 μM EGCG was the most potent dosage to extend lifespan via inducing a *mitohormetic* response via AMPK/SIRT1 and FOXO [11].

However, the poor bioavailability of green tea catechins in mammals [12, 13] makes it unlikely to achieve this concentration after oral administration in humans. Nevertheless, several independent clinical trials confirmed that green tea consumption improves various health parameters [1–4]. After administration of a maximum of 4.5 g of decaffeinated green tea solids, maximum plasma concentrations of EGCG, ECG, and EC reached in total roughly 2.5 μM in humans [14]. Consequently, we tested whether 2.5 μM is still sufficient to promote lifespan by inducing a *mitohormetic* response in *C. elegans.* In this work, we reveal that EGCG and ECG enhance fitness and increase the lifespan of *C. elegans* already at a concentration of 2.5 μM. This comparably low dosage is sufficient to inhibit the mitochondrial respiration chain activity in *C. elegans.* Experiments in isolated murine liver mitochondria revealed that EGCG and ECG hamper complex I activity. Inhibition of complex I was accompanied by transient ROS formation and an ATP drop after 6 h of EGCG and 12 h of ECG treatment in *C. elegans*. Lifespan extension of *C. elegans* by EGCG and ECG proved to be dependent on the presence of the energy sensors AMP-activated kinase AAK-2 and NAD-dependent protein deacetylase SIR-2.1, the homologs of mammalian AMPK and SIRT1, as well as on the ROS-sensing mitogen-activated protein kinase PMK-1, the orthologue of mammalian mitogen-activated protein kinase p38 MAPK, and in the following on its downstream targets protein skinhead-1 (SKN-1), the orthologue of nuclear factor erythroid 2-related factor 2 (NRF2), and DAF-16, the orthologue of a mammalian forkhead transcription factor (FOXO). These data suggest that the subsequent energy deficiency due to transient AMP drop triggers the energy sensors AAK-2 and SIR-2.1 in *C. elegans*. Moreover, the temporary increase in ROS levels might boost PMK-1 activity and, thereby, the respective signaling cascade, including SKN-1 and DAF-16 in *C. elegans.* Consistent with the concept of *mitohormesis*, these signaling pathways provoked an adaptive response by enhancing the activity of ROS defense enzymes superoxide dismutase (SOD) and catalase (CTL), increasing oxidative stress resistances, health, and lifespan. Moreover, metabolism changed in the long term, causing significantly reduced fat content in *C. elegans.* Taken together, inhibition of mitochondrial complex I once again proved to be a powerful tool to stimulate lifespan extension pathways.

## RESULTS

### EGCG and ECG promote lifespan, fitness, and stress resistance when applied at low doses

**Figure 1:**
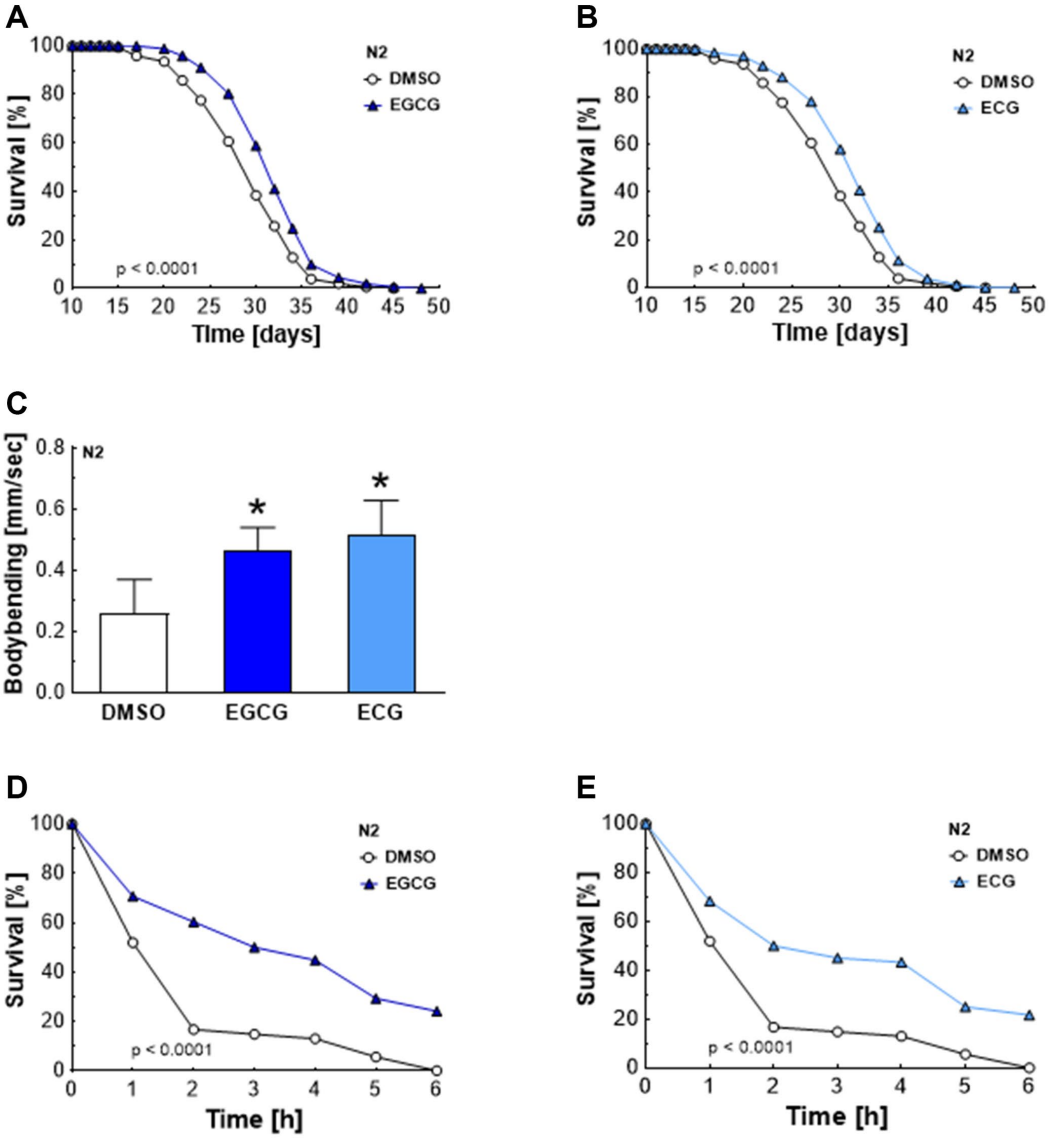
**Increased lifespan, locomotion activity, and stress resistance after EGCG and ECG treatment.** The representative outcome of lifespan assay of N2 wild-type nematodes in the presence of 2.5 μM EGCG versus DMSO. (**A**) The representative outcome of lifespan assay of N2 wild-type nematodes in the presence of 2.5 μM ECG versus control. (**B**) Locomotion quantification for N2 wild-type nematodes after 7 days exposure to DMSO, 2.5 μM EGCG, or 2.5 μM ECG. (**C**) The representative outcome of the survival analysis (h) of N2 nematodes in 50 mM paraquat solution after 7 days of pretreatment with EGCG (**D**) or ECG (**E**) in comparison to worms pretreated with DMSO. *P*-values are as indicated in the graphs. See Table 1 and Table 2 for corresponding detailed data and statistical analyses of lifespan assays and of paraquat stress assay, respectively.

Oral absorption and absolute bioavailability of green tea catechins are low in mammals [12], reaching total maximum plasma concentrations of 2.5 μM in humans after administration of maximal 4.5 g of decaffeinated green tea solids [14]. However, several independent clinical trials reported beneficial effects of EGCG and ECG regarding health parameters [1–4]. Therefore, we hypothesized that lower concentrations of EGCG and ECG than those studied previously [11] are still effective and improve lifespan and stress resistance in *C. elegans.* Indeed, EGCG and ECG applied at a concentration of 2.5 μM were sufficient to significantly extend the medium lifespan (Table 1) of *C. elegans* from 28.8 ± 0.3 to 30.8 ± 0.1 days (Figure 1A) and from 28.8 ± 0.3 to 30.6 ± 0.3 days (Figure 1B), respectively, causing an extension of 6.9% for EGCG and 6.2% for ECG treatment. The maximum lifespan (Table 1) was extended from 35.7 ± 0.6 to 36.9 ± 0.1 days by EGCG treatment (Figure 1A) and from 35.7 ± 0.6 to 37.1 ± 0.3 days by ECG treatment (Figure 1B), reaching an extension of 3.4% for EGCG and 3.9% for ECG. Next, we tested whether prolonged lifespan also correlates with improved fitness and stress resistance. Locomotion is dependent on functional muscle mass, connective tissues, and neuronal signaling. Consequently, motility is a suitable marker for health [15]. EGCG and ECG treatment improved the nematodes’ motility after 7 days of incubation (Figure 1C). Moreover, treatment of *C. elegans* with ECGC (Figure 1D) and ECG (Figure 1E) for 7 days significantly increased stress resistance (Table 2) to the free radical generator paraquat. Consequently, EGCG and ECG enhanced fitness and stress resistance, both crucial parameters for health.

**Table 1:** Lifespan results and statistical analysis.

**Strains, Compounds**	**Max lifespan in days ± SD (10^th^ percentile)**	**Medium lifespan in days ± SD (50^th^ percentile)**	**Number of experiments (*n*)**	***P* value versus control**	**Number of nematodes**
**N2 DMSO**	35.7 ± 0.6	28.8 ± 0.3	18		2831
**N2 EGCG**	36.9 ± 0.1	30.8 ± 0.1	18	<0.0001	2842
**N2 ECG**	37.1 ± 0.3	30.6 ± 0.3	15	<0.0001	2777
					
**N2 BHA**	36.4 ± 0.6	28.9 ± 0.4	9	0.3838	1548
**N2 BHA + EGCG**	36.0 ± 0.3	29.2 ± 0.4	9	0.3114	1581
**N2 BHA + ECG**	35.9 ± 0.5	29.6 ± 0.4	6	0.6682	1451
					
***aak-2 (ok524)* DMSO**	24.2	20.9 ± 0.2	3		462
***aak-2 (ok524)* EGCG**	24.4 ± 0.2	20.5 ± 0.3	3	0.1015	465
***aak-2 (ok524)* ECG**	24.6 ± 0.7	20.4 ± 0.4	3	0.9876	452
					
***sir-2.1 (ok434)* DMSO**	28.6 ± 0.1	24.3 ± 0.4	3		400
***sir-2.1 (ok434)* EGCG**	28.9 ± 1.2	24.5 ± 0.3	3	0.1858	355
***sir-2.1 (ok434)* ECG**	28.2 ± 0.5	23.7 ± 0.1	3	0.24	436
					
***pmk-1 (km25)* DMSO**	34.9 ± 0.6	27.9 ± 0.4	3		548
***pmk-1 (km25)* EGCG**	35.5 ± 1.5	28.2 ± 1.1	3	0.7759	567
***pmk-1 (km25)* ECG**	35.3 ± 0.7	28.0 ± 0.3	3	0.7363	581
					
***skn-1 (zu67)* DMSO**	16.5 ± 0.5	14.1 ± 0.1	6		424
***skn-1 (zu67)* EGCG**	17.0 ± 0.2	14.2 ± 0.1	6	0.5286	432
***skn-1 (zu67)* ECG**	16.8 ± 0.2	14.3	6	0.4823	440
					
***daf-16 (mgDF47)* DMSO**	22.5 ± 0.3	20.1 ± 0.1	3		660
***daf-16 (mgDF47)* EGCG**	22.2 ± 0.2	19.8	3	0.029	774
***daf-16 (mgDF47)* ECG**	21.7 ± 0.6	19.4 ± 0.2	3	<0.0001	707
					
***sod-2 (gk257)* DMSO**	33.3 ± 0.4	27.2 ± 0.4	3		730
***sod-2 (gk257)* EGCG**	33.1 ± 0.4	27.5 ± 0.4	3	0.9525	788
					
***ctl-2 (ok1137)* DMSO**	32.5 ± 0.4	26.8 ± 0.7	3		602
***ctl-2 (ok1137)* ECG**	32.9 ± 0.5	27.2 ± 0.4	3	0.1718	614

**Table 2:** Paraquat stress assay results and statistical analysis.

**Treatments**	**Number of experiments (*n*)**	***P* value versus control**	**Number of nematodes**
**N2 DMSO**	6		94
**N2 EGCG**	6	<0.0001	92
**N2 ECG**	6	<0.0001	93

### Complex I inhibition by EGCG and ECG hampers mitochondrial respiration temporarily and induces a transient ROS signal

**Figure 2:**
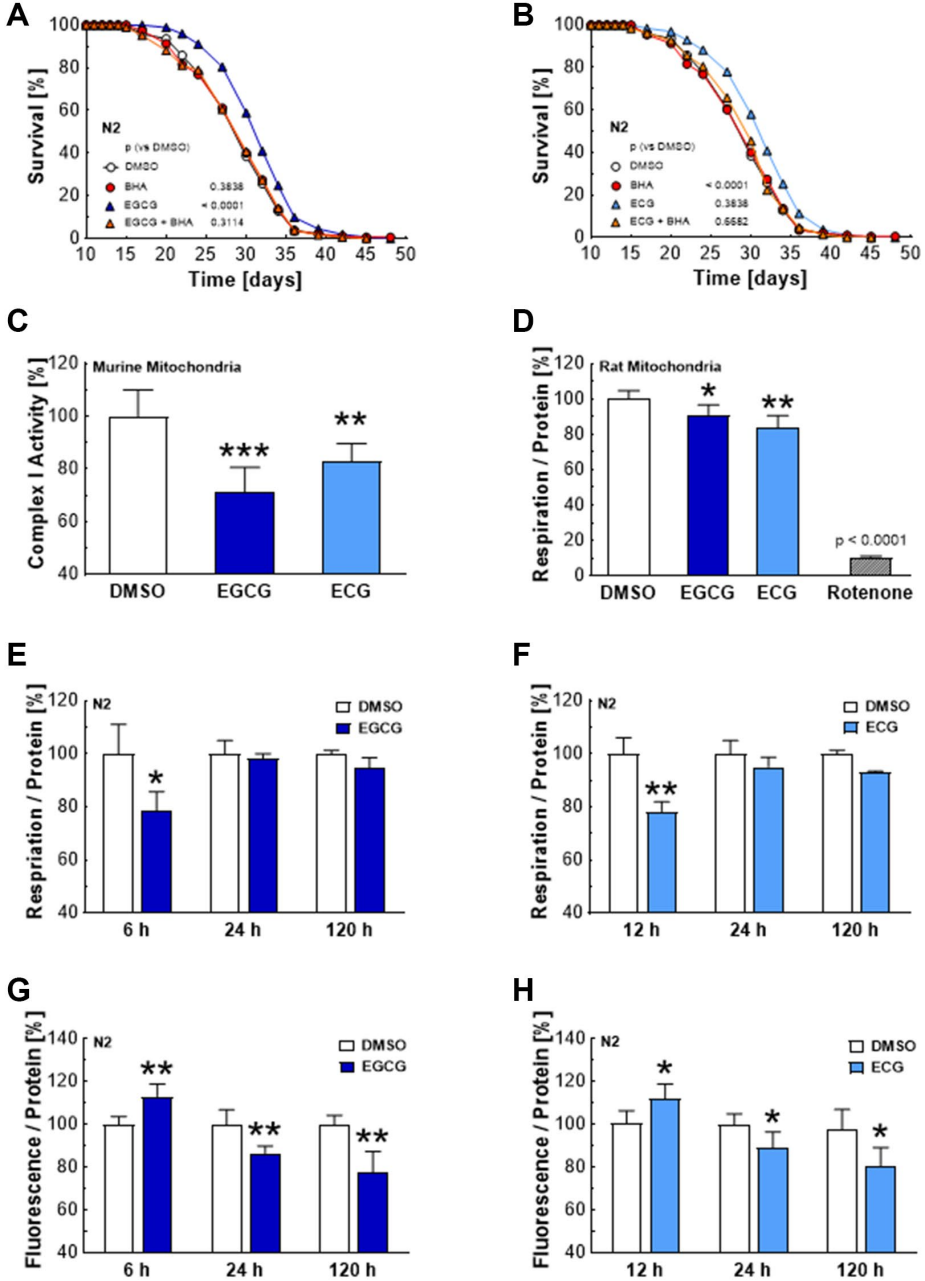
**EGCG and ECG inhibit complex I, which results in a temporary hampering of mitochondrial respiration and a boost in ROS production.** The representative outcome of lifespan assay of N2 wild type nematodes in the presence of 2.5 μM EGCG co-applied with an antioxidant; DMSO vs. 2.5 μM EGCG vs. 10 μM BHA vs. 2.5 μM EGCG in combination with 10 μM BHA. (**A**) The representative outcome of lifespan assay of N2 wild type nematodes in the presence of 2.5 μM ECG co-applied with an antioxidant; DMSO vs. 2.5 μM ECG vs. 10 μM BHA vs. 2.5 μM ECG in combination with 10 μM BHA. (**B**) Complex I activity in murine liver mitochondria after treatment with DMSO, 25 μM EGCG or 25 μM ECG. (**C**) Mitochondrial respiration of rat liver mitochondria after treatment with DMSO, 25 μM EGCG or 25 μM ECG. (**D**) Mitochondrial respiration of N2 wild-type nematodes after treatment with DMSO or 2.5 μM EGCG for 6 h, 24 h, or 120 h measured as oxygen consumption rate and normalized to protein content. (**E**) Mitochondrial respiration of N2 wild-type nematodes after treatment with DMSO or 2.5 μM ECG for 12 h, 24 h, or 120 h, measured as oxygen consumption rate and normalized to protein content. (**F**) ROS production of N2 wild-type nematodes after treatment for 6 h, 24 h, or 120 h with 0.1% DMSO or 2.5 μM EGCG. (**G**) ROS production of N2 wild-type nematodes after treatment for 6 h, 24 h, or 120 h with 0.1% DMSO or 2.5 μM ECG. (**H**) *P*-values are as indicated in the graphs. See Table 1 for corresponding detailed data and statistical analyses of lifespan assays.

Previous reports have suggested that green tea catechins induce SIRT1/SIR-2.1 and FOXO/DAF-16 signaling by an initial increase in ROS levels. However, the ROS source has remained unidentified in previous reports [11]. We could confirm that ROS is essential for lifespan extension provoked by catechins, showing that the antioxidant butylated hydroxyanisole (BHA) prevents the life-prolonging effect of ECGC (Figure 2A) and ECG (Figure 2B). Moreover, we found that 25 μM of EGCG and ECG significantly hamper the activity of complex I in murine liver mitochondria (Figure 2C) and the mitochondrial respiration in mitochondria isolated from rat liver (Figure 2D). These findings are in line with reduced mitochondrial respiration in *C. elegans* after 6–12 hours exposure to 2.5 μM EGCG (Figure 2E) or ECG (Figure 2F). Notably, mitochondrial respiration recovered after 24 h and 120 h of treatment with EGCG (Figure 2E) and ECG (Figure 2F), pointing to compensation of an initially impaired mitochondrial function. The time course of initial diminution and the subsequent recovery of mitochondrial respiration correlates with ROS levels, which increased significantly after 6 h of ECGC (Figure 2G) and 12 h of ECG (Figure 2H) administration and dropped significantly after 24 h and 120 h of catechin treatment (Figure 2G, 2H).

### AMPK and SIRT1 are essential for catechin-induced lifespan extension

**Figure 3:**
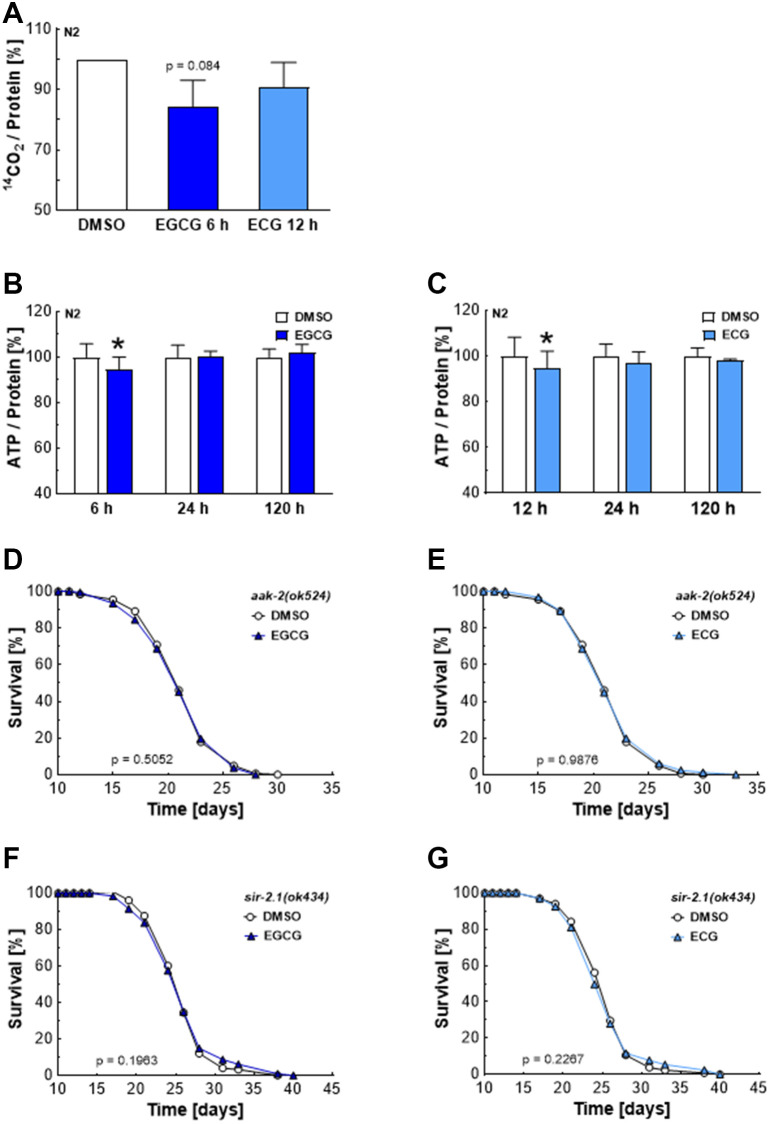
**EGCG and ECG induce a drop in cellular ATP levels and require AMPK/SIRT1 signaling to extend lifespan.** ^14^CO_2_ production of N2 wild-type nematodes after treatment with 0.1% DMSO, 2.5 μM EGCG or 2.5 μM ECG for the indicated time. (**A**) ATP content for various incubation periods of N2 wild-type nematodes with 0.1% DMSO or 2.5 μM EGCG. (**B**) ATP content for different incubation periods of N2 wild-type nematodes with 0.1% DMSO or 2.5 μM EGCG. (**C**) The representative outcome of lifespan assay of *aak-2* mutants treated with 0.1% DMSO versus 2.5 μM EGCG (**D**) or 2.5 μM ECG. (**E**) The representative outcome of lifespan assay of *sir-2.1* mutants treated with 0.1% DMSO versus 2.5 μM EGCG (**F**) or 2.5 μM ECG. (**G**) *P*-values are as indicated in the graphs. See Table 1 for corresponding detailed data and statistical analyses of lifespan assays.

Inhibition of complex I reduces NADH’s oxidation to NAD+, necessary for glyceraldehyde 3-phosphate conversion to 1, 3-bisphosphoglycerate during glycolysis. Consequently, reduced levels of NAD+ hamper glycolysis and the production of pyruvate, which enters the Krebs cycle to be converted into water and CO_2_ [16]. In line with these reports, ECGC reduced the oxidation of radioactively labeled glucose by 20%, as shown by impaired production of the ^14^C-labeled CO_2_ (Figure 3A). ECG treatment also tended to reduce the glucose turnover. However, the effects remained non-significant (Figure 3A). The time course of metabolic manipulation by EGCG and ECG was also reflected in overall ATP levels. In line with catechin-induced inhibition of mitochondrial respiration (Figure 2E, 2F) and glycolysis (Figure 3A), overall ATP levels dropped after 6 h of EGCG (Figure 3B) and 12 h of ECG (Figure 3C) treatment in nematodes before recovering after 24 h. A lack of ATP, resulting in a higher AMP to ATP ratio, is well-known to activate the AMP-dependent kinase AMPK [17]. The *C. elegans* homolog of AMPK, AAK-2, is involved in lifespan extension in response to impaired glycolysis [18] and insulin/IGF-1 signaling [19]. Indeed, EGCG (Figure 3D) and ECG (Figure 3E) failed to extend lifespan in *aak-2* deficient mutants. Notably, AMPK enhances NAD+-dependent type III deacetylase sirtuin 1 activity by increasing cellular NAD+ levels [20]. In *sir-2.1* defective mutants, EGCG (Figure 3F) and ECG (Figure 3G) did not achieve a lifespan extension, proving that EGCG and ECG prolong lifespan in an AMPK- and SIRT1-dependent manner. These findings align with previous reports showing that catechins’ lifespan extension depends on AMPK, SIRT1, and FOXO [11].

### p38 MAPK, NRF2, and FOXO are required for the lifespan extension induced by catechins

**Figure 4:**
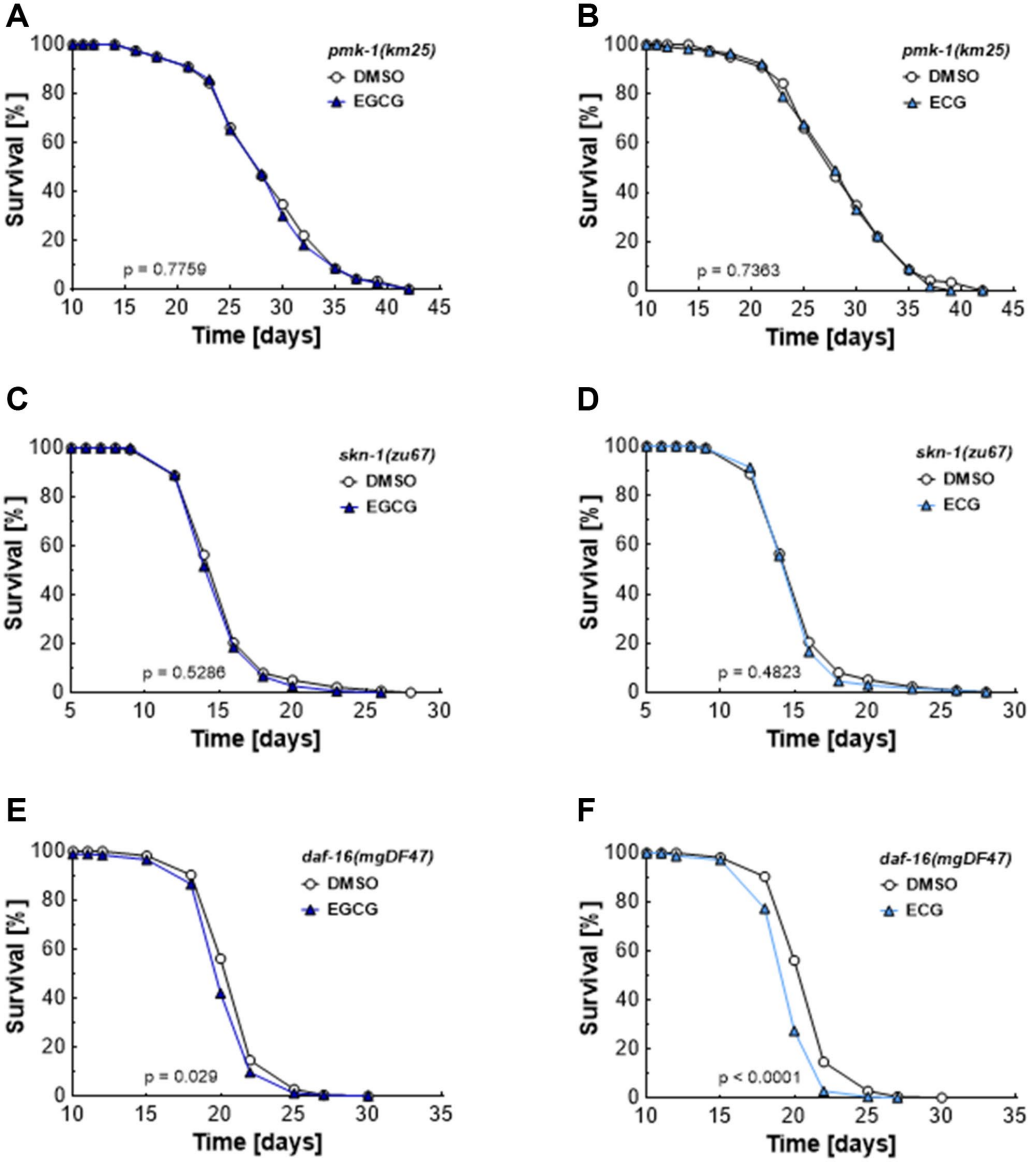
**EGCG and ECG mediate lifespan extension dependent on PMK-1/p38 MAPK, SKN-1/NRF2, and DAF-16/FOXO.** The representative outcome of lifespan assay of *pmk-1* mutants treated with 0.1% DMSO versus 2.5 μM EGCG (**A**) or 2.5 μM ECG. (**B**) The representative outcome of lifespan assay of *skn-1* mutants treated with 0.1% DMSO versus 2.5 μM EGCG (**C**) or 2.5 μM ECG. (**D**) The representative outcome of lifespan assay of *daf-16* mutants treated with 0.1% DMSO versus 2.5 μM EGCG (**E**) or 2.5 μM ECG. (**F**) *P*-values are as indicated in the graphs. See Table 1 for corresponding detailed data and statistical analyses of lifespan assays.

As shown in Figure 2, EGCG and ECG block complex I activity and, thus, induce a transient rise in ROS levels. ROS [21] and AMPK [22] are potential mediators of the p38 MAP kinase pathways. The homolog of the mammalian p38 MAPK, PMK-1, has been identified as a crucial component in the lifespan extension of *C. elegans* [23, 24]. In line with these previous reports, we found that neither EGCG (Figure 4A) nor ECG (Figure 4B) treatment extends lifespan in *pmk-1* deficient mutants. Next, we tested the impact of whether the transcription factor SKN1, the worm homolog of NRF2 and a downstream target of PMK1 under conditions of oxidative stress [25–27], is involved in the lifespan extension provoked by catechins. Again, no EGCG- (Figure 4C) or ECG-induced (Figure 4D) lifespan extension could be observed in *skn-1* mutant worms. DAF-16 is the homolog of a mammalian FOXO and is reported to respond to physical and environmental stress [28]. *daf-16* mutant worms are sensitive to oxidative stress and have shortened lifespans. Moreover, DAF-16 can activate or repress the transcription of target genes involved in dauer formation, life span, stress resistance, and fat storage of *C. elegans* [29]. EGCG and ECG decreased mean lifespan in *daf-16* deficient nematodes from 20.1 ± 0.1 to 19.8 days (Figure 4E) and from 20.1 ± 0.1 to 19.4 ± 0.2 days (Figure 4F), respectively. The maximum lifespan was decreased from 22.5 ± 0.3 to 22.2 ± 0.2 days by EGCG treatment (Figure 4E) and from 22.5 ± 0.3 to 21.7 ± 0.6 days by ECG treatment (Figure 4F). These results suggest that DAF-16 is indispensable for EGCG’s and ECG’s lifespan extension and show that *daf-16* deficient nematodes are especially prone to a ROS level rise induced by catechins.

### EGCG and ECG induce adaptive responses in ROS homeostasis and cellular metabolism

**Figure 5:**
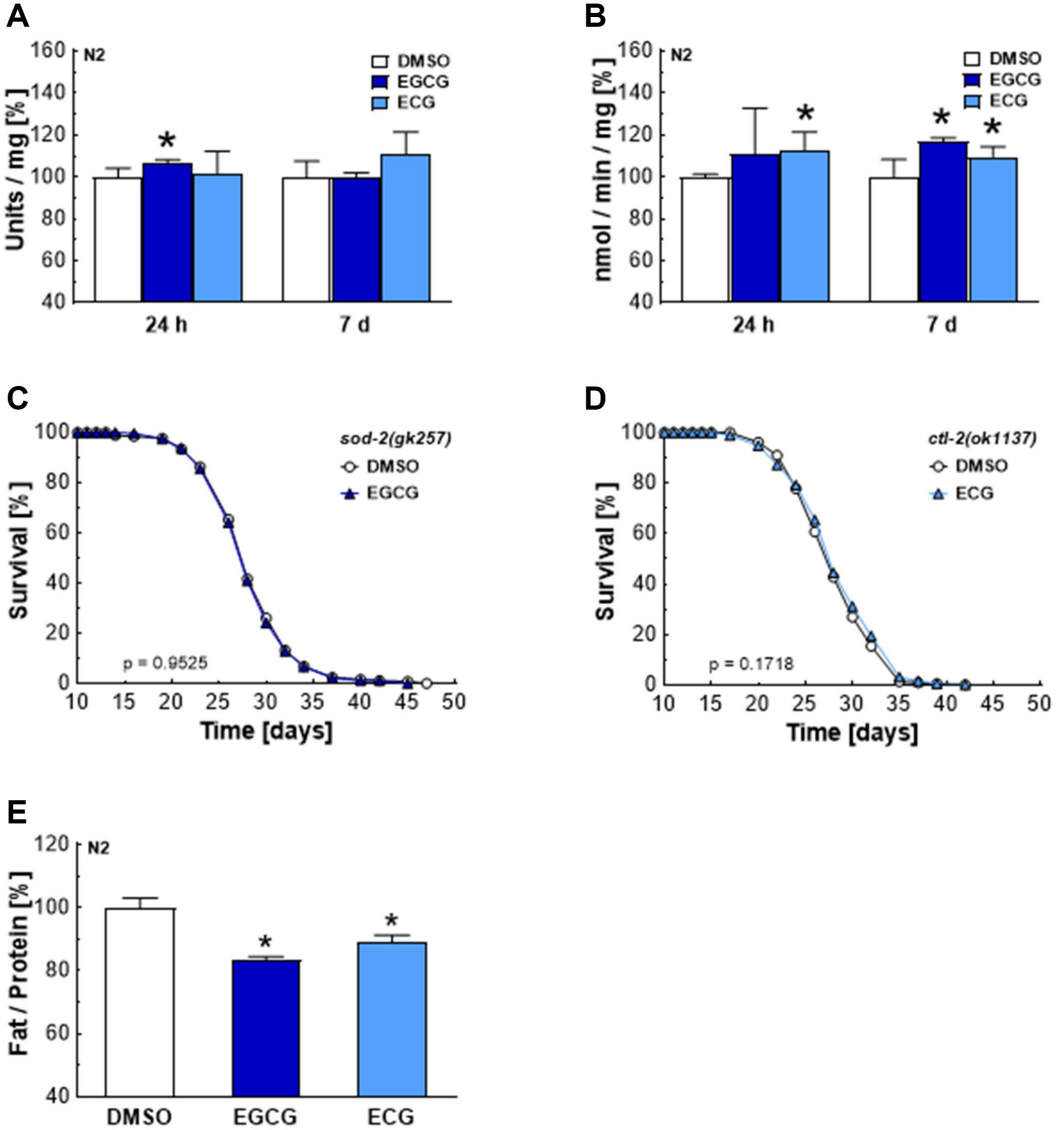
**EGCG and ECG induce SOD and CTL activity and a shift in lipid metabolism in the long term.** SOD (**A**) or CTL (**B**) activity after treatment with 0.1% DMSO, 2.5 μM EGCG or 2.5 μM ECG for 24 h or 7 days. The representative outcome of lifespan assay of *sod-2* mutants treated with 0.1% DMSO or 2.5 μM EGCG. (**C**) The representative outcome of lifespan assay of *ctl-2* mutants treated with 0.1% DMSO or 2.5 μM ECG. (**D**) Triglyceride content in N2 wild-type nematodes after treatment with 0.1% DMSO, 2.5 μM EGCG or 2.5 μM ECG for 5 days, normalized to protein content. (**E**) *P*-values are as indicated in the graphs. See Table 1 for corresponding detailed data and statistical analyses of lifespan assays.

AMPK/SIRT1 and p38MAPK/NRF2/FOXO signaling cascades are associated with antioxidant defense mechanisms [30]. The major antioxidant enzymes in *C. elegans* include five distinct superoxide dismutases, converting superoxide to hydrogen peroxide, and two catalases, which ensure the subsequent conversion of hydrogen peroxide to water [31]. EGCG treatments increased SOD activity after 24 h (Figure 5A) and CTL activity after 7 days (Figure 5B). Meanwhile, ECG treatments did not significantly increase SOD activity (Figure 5A) but increased CTL activity after 24 h and 7 days (Figure 5B). The enhanced activity of SOD and CTL correlates with the subsequent drop of ROS levels after 24 h of EGCG and ECG treatment. Notably, the lifespan-extending effect of EGCG and ECG is dependent on SOD-2 (Figure 5C) and catalase 2 (CTL-2) (Figure 5D). As shown in Figure 3, complex I inhibition by EGCG and ECG was also accompanied by a reduction in glucose oxidation. In line with this finding, the fat content was found to be significantly lower after 120 h of EGCG or ECG treatment (Figure 5E), pointing to a catechin-induced long-term reprogramming of cellular metabolism.

## DISCUSSION

Green tea is one of the most widely consumed beverages worldwide [32]. The popularity of green tea makes it crucial to study its impact on health and aging. Although EGCG’s and ECG’s bioavailability is relatively low [7, 8], consuming 4 cups of green tea daily for 8 weeks significantly decreases body weight [33]. Previous reports already reported a lifespan extension in *C. elegans* after treatment with 50–300 μM EGCG [11]. Here, we show that already 2.5 μM of EGCG and ECG, a concentration also potentially achieved after green tea consumption [14], are sufficient to induce an extension of lifespan and increase stress resistance by adaptational mechanisms. In this mitohormetic response, EGCG and ECG act initially as prooxidants by provoking a ROS rise. Since a transient ROS burst induces antioxidant defense mechanisms, EGCG and ECG display antioxidant properties in the long term. In higher concentrations, EGCG and ECG might show harmful effects due to excessive ROS production. This phenomenon gets obvious in studies performed on cancer cells. While the antioxidant potential of green tea catechins in low concentrations was suggested as a potential solution to prevent tumorigenesis [34, 35], higher dosages of catechins might serve as antitumor agents due to the induction of overwhelming ROS formation and apoptosis [36–41]. Notably, EGCG was more potent than ECG in human cancer cell lines in inducing cytotoxic effects [33] and inhibiting cancer cell motility [42]. Indeed, it took just 6 h for EGCG, but 12 h for ECG to affect mitochondrial respiration, ROS, and ATP levels. However, the impact of these compounds was similar when applied in the long term, yielding similar effects on lifespan, motility, and stress resistance.

Besides triggering a mitohormetic response through their effects on transcription factors and enzyme activities, catechins were speculated to exert direct antioxidant potential by scavenging ROS [43, 44]. While a modest increase in the plasma antioxidant capacity following green tea consumption was reported [43], the fraction of structurally intact catechins reaching target tissues is insignificant compared to the antioxidant potential due to intracellular glutathione achieving levels of 1–11 mM [45–47]. Besides, EGCG even induced hydrogen peroxide formation in the cell culture and liquid NGM system [44–46]. Moreover, hydrogen peroxide mimicked the effect of EGCG on signaling pathways, while antioxidants abolished the impact of catechins [37, 41, 48–50]. We could show that BHA prevented lifespan extension by EGCG and ECG, suggesting that an initial rise in ROS levels is necessary to induce adaptational mechanisms causing improved antioxidant properties.

Previous studies already revealed increased hydrogen peroxide levels and a dose- and time-dependent decrease in glutathione levels in cell culture models after applying 50 μM of EGCG [43, 51]. However, the mechanism of how EGCG and ECG induce ROS formation was not described so far [11]. In the current study, we revealed that EGCG and ECG inhibit complex I of the ETC. Experiments in rat cerebellar granule neurons have shown that EGCG accumulates explicitly in mitochondria, reaching 90–95% mitochondrial accumulation of this polyphenol [52]. This finding is well aligned with the plethora of literature describing polyphenols as compounds targeting mitochondria [53, 54]. Consequently, we isolated mitochondria to investigate the impact of EGCG and ECG on the complexes of the mitochondrial ETC. Isolated mitochondria are separated from their natural environment and signaling processes, and the isolation process brings the risk of damaging mitochondrial membranes due to shear forces [55]. However, drug uptake by mitochondria is dependent on the integrity of the outer and inner mitochondrial membrane, including the function of transporter proteins and carriers [56]. Since 25 μM of EGCG and ECG were necessary to achieve a significant inhibition of complex I activity in mitochondria isolated from murine liver samples and to hamper mitochondrial respiration in mitochondria isolated from rat liver, we assume that the isolation process affected the integrity of mitochondrial membranes and, thereby, mitochondria’s potential to take up catechins efficiently.

Besides, the isolation of mitochondria yields a relatively homogenous population of spherical organelles with disorganized cristae and diluted matrix content. The structural alterations affect ETC activity and mitochondrial respiration rate [57]. We assume that structural changes in cristae organization due to the isolation process might be another reason why 25 μM of EGCG and ECG were necessary to significantly block complex I activity and mitochondrial respiration rate in isolated mitochondria.

In addition, we present that a temporary hampered mitochondrial respiration goes along with a transient rise in ROS levels and a brief drop in ATP, triggering signaling pathways associated with lifespan extension in *C. elegans*. Our findings align with reports about the *C. elegans* mutant *nuo-6(qm200)*, carrying a mutation in a conserved subunit of mitochondrial complex I (NUDF84). This specific mutant has reduced complex I function, increased ROS levels [58], and a prolonged lifespan [59]. It was also speculated that blockage of the complex I of the mitochondrial electron transport chain delays aging due to slowed embryonic development and larval growth, decreased pumping and defecation rate, or a reduced accumulation of ROS damage [60–62]. However, RNAi-induced knockdown of the mitochondrial electron transport chain’s complexes at the L3/L4 stage is sufficient to initiate lifespan extension in *C. elegans*. At this stage, mitochondria are already undergoing a period of dramatic proliferation and massive mitochondrial DNA expansion [63]. Moreover, inhibiting respiratory chain components during adulthood did not provoke lifespan extension anymore [64–66]. Consequently, one has to assume that a temporary sub-lethal rise in mitochondrial ROS during early adulthood induces lifespan extension by provoking changes in the homeostasis of proteins [59, 67] and metabolism [58]. Notably, glucose restriction by 2-deoxy-D-glucose (2-DG)-mediated inhibition of glycolysis increases the lifespan in *C. elegans* in a ROS-dependent manner [18], suggesting that the temporary drop in ATP levels due to complex I inhibition is an additional trigger to prolong lifespan.

Our data demonstrate that life span extension by EGCG and ECG involves energy sensors AAK-2/AMPK and SIR-2.1/SIRT1 as well as the ROS-sensing PMK-1/p38 MAPK, and the transcription factors SKN-1/NRF2 and DAF-16/FOXO. By activating these signaling cascades, the function of ROS defense enzymes, SOD and CTL, and the oxidative stress resistance gets boosted. A previous report presented that catechins’ lifespan extension depends on AMPK, SIRT1, and FOXO [11]. Ahead of this report, SOD-3, DAF-16, and SKN-1 were already suggested as targets of EGCG due to enhanced expression [68] or translocation into the nucleus after respective compound treatment [48]. Oxidative stress was reported to stimulate SKN-1’s translocation to the nucleus, a process tightly regulated by protein kinases, including PMK-1, GSK-3, MKK-4, IKK epsilon-1, NEKL-2, and PDHK-2 [26]. Notably, SKN-1 activation in neurons is necessary for dietary restriction-mediated lifespan extension [69]. Moreover, reduced insulin/IGF-1 signaling causes nuclear accumulation of SKN-1, a process needed for long-lived *daf-2* mutants with increased stress resistance and lifespan [19, 70]. DAF-16, the orthologue of mammalian FOXO, is a crucial regulator of longevity, metabolism, and dauer diapauses in *C. elegans* [28, 29, 71, 72]. Consequently, it seems reasonable that the ROS-sensing p38 MAPK and the energy-sensing AMPK activate the respective signaling cascades after blockage of complex I by EGCG and ECG. Reports showed that AMPK activates p38 MAPK [73]. Consequently, these two kinases might even augment each other’s activity and the potential of the respective signaling cascade.

The long-term effects also included reduced fat content in *C. elegans* after 5 days of catechin treatment. Align with this finding, inhibition of complex I and complex IV by rotenone and NaN3 reduced lipid accumulation in 3T3-L1 cells [74]. Moreover, a previous report revealed reduced body fat content in *C. elegans* after catechin treatment [75]. Besides, green tea catechins were associated with reduced obesity in zebrafish [76], mice [77], rats [78, 79], and humans [80, 81], suggesting a catechin-induced metabolic remodeling.

Clinical trials have already confirmed the safety of EGCG [7] and highlighted the potential in counteracting age-related cardiovascular and metabolic diseases [1–4]. Experiments in rodents studying physical and clinical parameters over time and further clinical trials are required to identify the best timing and dosage for administering catechins. Finally, these studies might characterize additional effects and downstream mechanisms of complex I inhibition. Despite the promising results obtained in animal experiments, the low bioavailability of EGCG [7] still raises the question of whether green tea catechins can reliably provoke beneficial effects in humans. Consequently, additional efforts might be needed to identify complex I inhibitors with increased bioavailability.

## CONCLUSIONS

**Figure 6:**
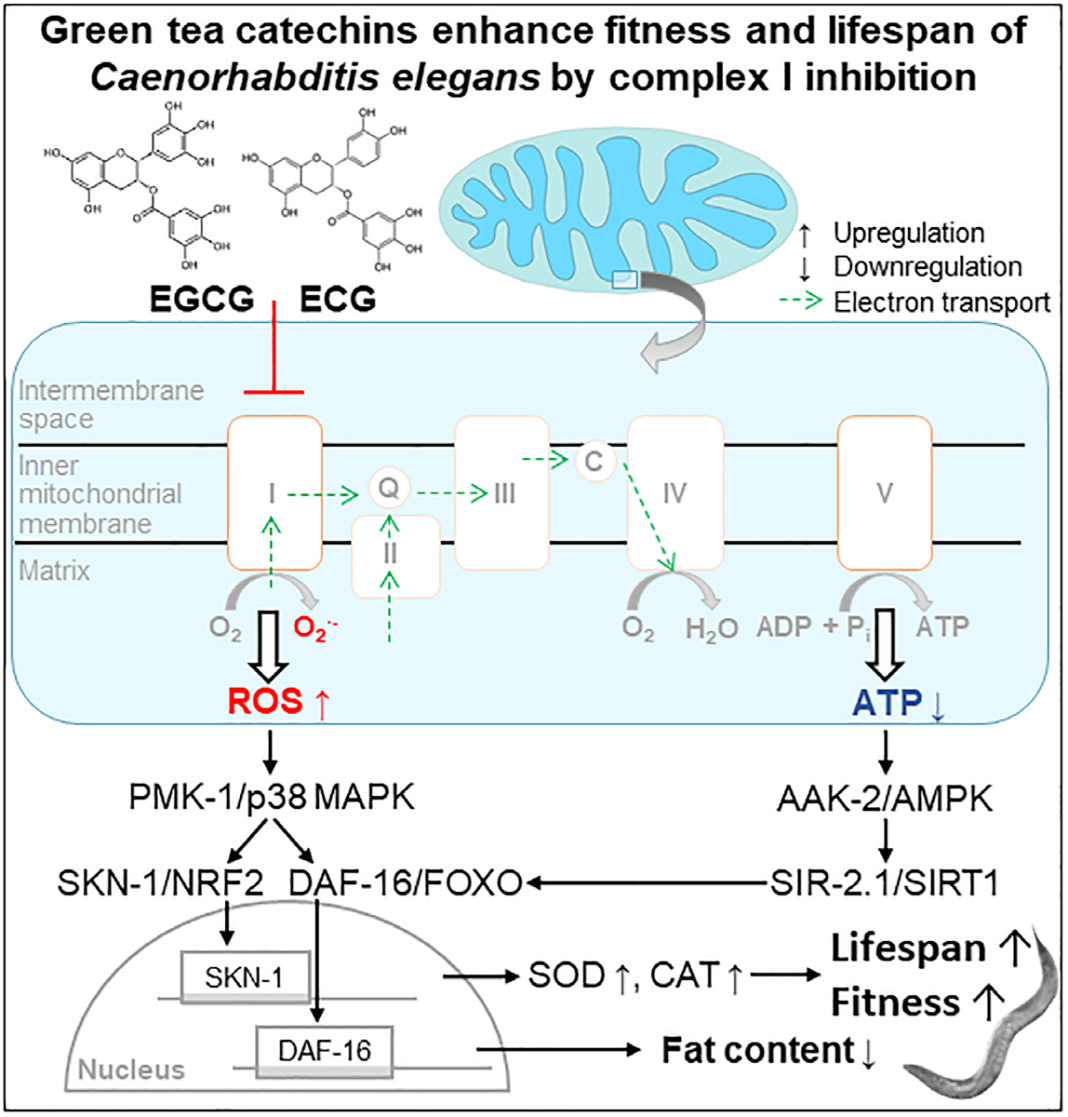
Scheme. Green tea catechins enhance fitness and lifespan of Caenorhabditis elegance by complex I inhibition.

We conclude that applying the green tea catechins EGCG and ECG at a low dose extends the lifespan of *C. elegans* via inducing a mitohormetic response. Thereby, the inhibition of complex I causes a transient ROS rise that stimulates the antioxidant defense enzymes SOD and CAT and activates the PMK-1/SKN-1/DAF-16 pathway (Figure 6, Scheme). Besides, complex I inhibition causes a temporary drop in cellular ATP levels and consequently activation of AAK-2/SIR-2.1 signaling. In the long term, the re-wiring of these energy- and ROS-dependent pathways reduces the fat content and extends health- and lifespan.

## METHODS

### Nematode strains and maintenance

*C. elegans* strains used in the current study were obtained from the *Caenorhabditis* Genetics Center (CGC, University of Minnesota). Nematodes were grown and maintained at 20°C in 10 cm Petri dishes on nematode growth media (NGM), with *Escherichia coli (E. coli)* OP50 bacteria as the food resource as previously described [18, 82, 83]. The strains used in this study included the following: N2 (wild type), GA1001 *aak-2(ok524)*, VC199 *sir-2.1(ok434),* KU25 *pmk-1(km25)*, EU1 *skn-1(zu67),* IU10 *daf-16(mgDF47),* GA184 *sod-2(ok257)*, and VC754 *ctrl-2(ok1137).*

### Compound treatment

EGCG, ECG, and BHA dissolved in DMSO, reaching a stock concentration of 2.5 mM of EGCG and ECG and 10 mM of BHA. The NGM agar solution was autoclaved and subsequently cooled to 55°C, before supplements and compounds (EGCG, ECG, BHA, or DMSO) were added under continuous stirring. The final concentration for compounds was calculated regarding the volume of agar, and the same volume of DMSO was added to control plates. NGM agar plates were supplemented with 100 μg/ml ampicillin to induce metabolic inactivity in *E. coli*. Agar plates were poured and dried, sealed with parafilm, and stored at 4°C. Before experiments, NGM plates were spotted with a bacterial lawn of heat-inactivated bacteria (OP50 HIT) to avoid interference by a potential xenobiotic-metabolizing activity of *E. coli*. To exclude any effects on development, the incubation period with compounds started at the L4 stage by transferring nematodes to the respective NGM plates [84]. To analyze oxygen consumption rate, glucose oxidation, ATP levels, enzyme activity, and fat content, adult worms at the L4 stage were transferred on NGM agar plates containing 25 μM 5-fluoro-2′-deoxyuridine (Sigma-Aldrich, St. Louis, MO, USA) to prevent progeny formation. After 16 h, we transferred animals to respective treatment groups and harvested them at the indicated time points [18].

### Lifespan analyses

According to standard protocols, all lifespan assays were performed at 20°C as previously described [18, 19]. Briefly, the *C. elegans* population was synchronized with hypochlorite/NaOH solution except for *skn-1* mutant worms. Eggs from heterozygous *skn-1* hermaphrodites were harvested after overnight egg-laying without applying hypochlorite/NaOH solution to increase the yield of viable larvae [85]. Eggs of nematodes were transferred to NGM plates with fresh OP50 bacteria to allow hatching and development. After approximately 64 h, at the L4 stage, we moved 200 nematodes manually to freshly prepared NGM plates containing the respective compounds and supplied them with a lawn of OP50 HIT. During the first 10–14 days, nematodes were transferred to freshly prepared NGM treatment plates every day and later every second day. Nematodes without any reaction to gentle stimulation were classified as dead. Nematodes that crawled off the plate or suffered from non-natural death like internal hatching were censored and excluded from statistics on the day of premature death. Notably, for lifespan analysis using BHA, nematodes were propagated on BHA-containing NGM plates for four generations before synchronization; the same applied for the respective DMSO controls.

### Locomotion assay

Following the L4 stage, nematodes were treated with 0.1% DMSO, 2.5 μM of EGCG, and 2.5 μM of ECG for 7 days. Afterward, we transferred single worms into S-buffer containing 0.01% Triton X-100 to wash off bacteria and then pipetted them on a glass slide. Movements of single worms within the liquid system were recorded for 20 seconds by a digital CCD camera (Moticam 2300, Motic, St. Ingbert, Germany) coupled microscope (SMZ 168, Motic, St. Ingbert, Germany) equipped with Motic Images Plus 2. We analyzed the videos using the DanioTrack software (Loligo Systems, Tjele, Denmark), subtracting the background and determining the center of gravity of all object pixels compared to the background. As described previously, the center’s shift distance was accumulatively calculated and normalized per second [84].

### Paraquat stress resistance assay

Resistance to lethal oxidative stress by paraquat (Sigma-Aldrich, Munich, Germany) was assessed as previously described [18, 19]. Briefly, worms were treated with 0.1% DMSO, 2.5 μM of EGCG, and 2.5 μM of ECG for 7 days after L4 stage. Afterward, we transferred worms into 96-well plates: 6 nematodes in 100 μl of S-buffer, containing freshly dissolved 50 mM paraquat. Dead worms were scored every hour until all control worms were dead.

### Basal oxygen consumption rate

Mitochondrial respiration was quantified using a DW1/AD Clark-type electrode (Hansatech, King’s Lynn, England) as previously described [18]. Briefly, we treated worms with 0.1% DMSO, 2.5 μM EGCG, or 2.5 μM ECG for the indicated periods, then washed off the respective NGM plates with S-buffer and allowed them to settle by gravitation to remove offspring and bacteria. Worms were also washed twice with S-buffer and transferred into the DW1 chamber to monitor oxygen consumption for 10 mins. Afterward, we collected worms for Bradford protein determination [86].

### ROS quantification

Before the ROS measurement, MitoTracker Red CM-H2X ROS (Invitrogen, Carlsbad, CA, USA) incubation plates were prepared as previously described [19]. Briefly, we treated worms with 0.1% DMSO, 2.5 μM EGCG, or 2.5 μM ECG for the indicated periods, then washed off the respective NGM plates and allowed them to settle by gravitation to remove offspring and bacteria. Worms were additionally washed twice with S-buffer and transferred to freshly prepared MitoTracker Red CM-H2X incubation NGM plates containing 500 μl of OP50 HIT mixed with 100 μl freshly prepared MitoTracker Red CM-H2X stock solution (100 μM). After 2 h at 20°C, worms were washed off MitoTracker Red CM-H2X incubation NGM plates and transferred to NGM agar plates with 0.1% DMSO, 2.5 μM EGCG or 2.5 μM ECG for 1 h to remove excess dye from the gut. Aliquots of 100 μl worm suspension in S-buffer were distributed into a 96-well FLUOTRAC^™^ plate (Greiner Bio-One, Frickenhausen, Germany). Fluorescence intensity was measured on a microplate reader (FLUOstar Optima, BMG Labtech, Offenburg, Germany) using well-scanning mode (ex: 570 nm; em: 610 nm). We collected worms from plates for Bradford protein determination [86].

### Glucose oxidation assay

[^14^C] D-glucose oxidation rates were determined as described previously [87]. Uniformly labeled [^14^C] D-glucose was purchased from PerkinElmer, and the specific activity of the batch used was 300 mCi/mmol. We placed an equal number of nematodes on the NGM plates containing 0.1% DMSO, 2.5 μM EGCG, or 2.5 μM ECG for the indicated period. After collection and two subsequent washes in S-buffer, worm pellets were resuspended in the incubation buffer. 700 μl of the suspension were transferred to 4 cm Petri dishes. The latter were placed in 10 cm Petri dishes together with a second 4 cm Petri dish containing 600 μl of 0.1 M KOH solution to trap CO_2_ as described previously [18, 88]. Hence, each 10 cm dish was equipped with two 4 cm dishes, one carrying nematodes and the other containing KOH. We added labeled glucose to a final concentration of 17 μM U-[14C] D-glucose (5 μCi/ml) in the nematode suspension as a substrate. We added nonradioactive glucose into each sample to reach a final concentration of 0.5 mM. The 10 cm Petri dishes were covered, sealed with parafilm in an air-tight manner, and incubated at 20°C for 3 h. Subsequently, an aliquot of 500 μl of KOH was immersed in 4.5 ml of scintillation fluid and placed in a liquid scintillation counter (Beckmann LS 6000, Global Medical Instrumentation, Inc.) to quantify the amount of trapped ^14^CO_2_. We normalized ^14^CO_2_ signals to incubated worms’ protein content.

### ATP quantification

We treated nematodes with 0.1% DMSO, 2.5 μM EGCG, or 2.5 μM ECG for the indicated time. After collection and washing with S-buffer twice, worm pellets were shock frozen in liquid nitrogen and grinded in a nitrogen-chilled mortar. The grinded samples were boiled with 4 M Guanidine-HCl at 99°C for 15 min to destroy ATPase activity [58, 89]. Precipitated proteins were separated by centrifugation (30 min, 13200 g at 4°C), and the supernatant was analyzed regarding the ATP content using CellTiter Glo (Promega, Fitchburg, WI, USA) according to the manufacturer’s instructions. ATP values were normalized to protein content using the Bradford assay [86].

### Activity assays for Catalase (CTL) and Superoxide Dismutase (SOD)

After treating nematodes with 0.1% DMSO, 2.5 μM EGCG, or 2.5 μM ECG for the indicated period, the respective enzyme activities were determined by standard photometric assays as previously described [18, 19, 84]. Briefly, CTL activity was estimated by the production of formaldehyde due to the enzyme’s reaction with methanol in the presence of an optimal concentration of H_2_O_2_. The produced formaldehyde was determined spectrophotometrically with 4-amino-3-hydrazino-5-mercapto-1, 2, 4-triazole (Purpald, Applichem, Darmstadt, Germany). We measured SOD activity photometrically with a tetrazolium salt, forming a water-soluble formazan dye upon reduction with a superoxide anion.

### Fat content analysis

We determined fat content by applying a triglyceride determination kit (Roche, Mannheim, Germany) as previously described [18, 88] and normalized to protein content using the Bradford assay [86]. Briefly, worms were incubated with 0.1% DMSO, 2.5 μM EGCG, or 2.5 μM ECG for 5 days, washed, and shock-frozen in liquid nitrogen. Afterward, worm pellets were grinded in a nitrogen-chilled mortar with Milli-Q water supplemented with 5% Triton X-100 and sonicated 3 times. We centrifuged 200 μl of the homogenized extract and extracted the supernatant for protein determination. 400 μl of lysate was heated to 80°C for 5 min and then cooled down to room temperature. The heating was repeated once to dissolve all triglycerides. After heating and cooling, the lysate was centrifugated at 12000 *g* for 10 min, and we collected the supernatant for triglyceride determination according to the manufacturer’s protocol.

### Quantification of complex I activity in mitochondria from the murine liver

We measured the activity of complex I spectrophotometrically at 600 nm in 1 ml of 25 mM potassium phosphate buffer containing 3.5 g/L BSA, 60 μM 2,6-dichloroindophenol (DCIP), 70 μM decylubiquinone, 1.0 μM antimycin A, and 0.2 mM NADH, adjusted to pH 7.8 [90]. Decylubiquinone and antimycin A were dissolved in DMSO as 17.5 mM and 1.0 mM, respectively. DCIP and NADH were dissolved in water as 10 mM for both. BSA stock solution was 70 g/L in 5 mM potassium phosphate buffer, pH 7.4. Mouse liver mitochondria stocks contained 10 μg/μl in 10 mM Tris (pH 7.6) and were stored at −80°C. After being thawed, 30 μl of mitochondria were treated with 470 μl of 10 mM Tris-Cl, pH 7.6, to disrupt the mitochondrial membrane. Subsequently, 20 μl mitochondria fragments were preincubated in a 960 μl incubation mixture without NADH for 3 mins. After 3 mins, we added 20 μl of 10 mM NADH into the incubation mixture and measured the absorbance at 20 s intervals for 2 mins. 2 mins later, 1 μl of DMSO, EGCG, or ECG were added into the incubation mixture as fast as possible and measured absorbance again at 20s intervals for 4 mins. The effect of chemicals on complex I activity was expressed as the slopes’ ratio of decreasing absorbances before and after adding substances.

### Isolation of mitochondria from murine liver

We did the isolation of mitochondria from rat liver according to Frezza’s protocol [91], except for the homogenization, which was done using a tissue glass Dounce Homogenizer (Wheaton, VWR, Darmstadt, Germany). Briefly, rodents were fasted overnight and killed by cervical dislocation. The liver was rapidly explanted, immersed, and sliced in the isolation buffer containing 200 mM sucrose, 1 mM EGTA/Tris, and 10 mM Tris/MOPS, pH 7.4. The washed liver fragments were placed into the tube with around 25 ml isolation buffer. The loose-fitting pestle was inserted, pressed down, and lifted four times, and then the tight-fitting pestle was applied in the same way twice. The mixture was poured into the 50 ml polypropylene falcon tube and centrifuged at 600 *g* for 10 min at 4°C. We carefully removed the fat on the top of the supernatant by using tissue paper. The supernatant was extracted to a second polypropylene falcon tube centrifuged at 7000 *g* for 10 min at 4°C. Afterward, the fat was removed, the supernatant discarded, and the mitochondrial pellet resuspended in the remaining buffer. The suspension containing mitochondria was centrifuged again at 7000 *g* for 10 min at 4°C. The supernatant was removed entirely, and the mitochondrial pellet was resuspended in 200 μl isolation buffer as described above. The concentration of isolated mitochondria was determined with Bradford (1976).

### Quantification of oxygen consumption rate in murine liver mitochondria

Mitochondria respiration was quantified using a DW1/AD Clark-type electrode (Hansatech, King’s Lynn, England) at 30°C in 1 ml experiment buffer containing 125 mM KCl, 10 mM Tris/MOPS, 0.1 mM EGTA/Tris and 1 mM KH_2_PO_4_, pH 7.4, as previously described [91]. 5 mM Glutamate and 2.5 mM Malate were supplied as substrates for complex I, III, IV. After recording basal respiration for 2 min, 0.1% DMSO, 25 μM EGCG, or 25 μM ECG and subsequently 100 μM ADP was added. After ADP was wholly consumed, the oxygen consumption rate slowed down, 5 mM succinate, and ADP were added to study complex II, III, IV activity. At the end of each measurement 60 nM FCCP were supplied to check the viability of mitochondria.

### Statistical analyses

Data are expressed as means ±SD unless otherwise indicated. Statistical analyses for all data except lifespan assays and stress resistance assays were performed by Student’s *t-*test after testing for equal distribution of the data and equal variances within the data set. Statistical calculations were carried out using the log-rank test to compare significant distributions between the different groups in lifespan and stress resistance assays. We performed all analyses using Microsoft Office Excel 2016 (Microsoft, Albuquerque, NM, USA). Differences were considered statistically significant at *p* < 0.05 and presented as specific *p*-values (^*^p ≤ 0.05; ^**^p ≤ 0.01; ^***^p ≤ 0.001).
